# Supplementary material for: Celiac Disease and Liver Damage: The Gut–Liver Axis Strikes Back (Again)? A Retrospective Analysis in the Light of a Literature Review
Source: Nutrients. 2024 Dec 28;17(1):85. doi: 10.3390/nu17010085 (PMC11722968; doi:10.3390/nu17010085)
Supplement: Supplementary file 1 [file nutrients-17-00085-s001.zip › nutrients-3386793-supplementary.pdf]

Supplementary Table S1: Baseline (T0) demographic and clinical features of CD population

	N (160)
Age at CD diagnosis (median; IQR)	6.4 (0.8-13.2)
6 months – 1 year and 5 months (n, %)	8 (5.0)
1 year and 6 months – 4 years and 11 months (n, %)	75 (46.9)
5 years – 14 years and 11 months (n, %)	77 (48.1)
Sex	
Male (n, %)	56 (35.0)
Female (n, %)	104 (65.0)
Ethnicity	
Caucasian (n, %)	159 (99.4)
African (n, %)	1 (0.6)
BMI class:	
Underweight (n, %)	22 (13.8)
Healthy weight (n, %)	87 (54.4)
Overweight (n, %)	47 (29.4)
Obesity (n, %)	2 (1.2)
Severe obesity (n, %)	2 (1.2)
Familial history of CD (n, %)	39 (24.4)
CD type:	
Typical (n, %)	81 (50.6)
Atypical (n, %)	52 (32.5)
Silent (n, %)	27 (16.9)
Stool disorders:	
None (n, %)	96 (60.0)
Diarrhea (n, %)	29 (18.1)
Constipation (n, %)	18 (11.3)
Mixed bowel movements (n, %)	17 (10.6)
Abdominal pain (n, %)	57 (35.6)
Weight loss (n, %)	33 (20.6)
Growth retardation (n, %)	52 (32.5)
Autoimmune diseases (n, %)	28 (17.5)
Type 1 diabetes (n, %)	14 (8.8)
Autoimmune thyroiditis (n, %)	6 (3.8)
Type 1 diabetes and autoimmune thyroiditis (n, %)	5 (3.1)
Other (n, %)	3 (1.9)
Extraintestinal symptoms (n, %)	40 (25.0)
Hypertension (n, %)	1 (0.6)
Anemia (n, %)	36 (22.5)

BMI: Body Mass Index; CD: celiac disease; IQR: interquartile range;

Supplementary Table S2: Baseline (T0) laboratory, US and histological features of the CD population

	N (160)
Hb (g/dl) (mean ± SD)	12.1 (1.5)
MCV (fl) (mean ± SD)	73.5 (8.1)
MCH (pg) (mean ± SD)	24.9 (4.1)
RDW (%) (mean ± SD)	16.1 (5.1)
Blood iron (μg/dl) (mean ± SD)	62.3 (32.9)
Ferritin (ng/ml) (median; IQR)	18.6 (7.2-34.9)
Transferrin (mg/dl) (median; IQR)	322.0 (248.0-392.0)
WBC (10^3) (mean ± SD)	8.2 (3.9)
Platelets (10^3) (median; IQR)	317.0 (267.5-384.6)
AST (U/l) (mean ± SD)	26.9 (10.3)
ALT (U/l) (mean ± SD)	21.3 (8.3)
Hypertransaminasemia (n, %)	13 (8.1)
US liver steatosis (n, %)	6/99 (6.1)
Albumin (g/dl) (mean ± SD)	4.2 (0.5)
Total bilirubin (mg/dl) (mean ± SD)	0.3 (0.2)
Direct bilirubin (mg/dl) (mean ± SD)	0.1 (0.1)
ALP (U/l) (mean ± SD)	199.8 (75.0)
GGT (U/l) (mean ± SD)	9.2 (5.8)
Total cholesterol (mg/dl) (mean ± SD)	156.9 (26.0)
HDL (mg/dl) (mean ± SD)	56.6 (24.6)
LDL (mg/dl) (mean ± SD)	78.1 (29.3)
Triglycerides (mg/dl) (median; IQR)	69.0 (49.3-109.8)
Fasting blood glucose (mg/dl) (mean ± SD)	108.1 (68.0)
APRI>0.7 (n, %)	0 (0.0)
FIB-4>1.45 (n, %)	0 (0.0)
IgA (g/dl) (mean ± SD)	137.4 (73.0)
INR (mean ± SD)	0.8 (0.4)
TSH (U/ml) (mean ± SD)	2.3 (1.3)
fT3 (pg/ml) (mean ± SD)	3.8 (2.6)
fT4 (ng/dl) (mean ± SD)	1.8 (3.0)

ALP: alkaline phosphatase; ALT: alanine aminotransferase; APRI: AST to platelet ratio index; AST: aspartate aminotransferase; CD: celiac disease; FIB-4: fibrosis-4 index; fT3: triiodothyronine; fT4: thyroxine; GGT: gamma glutamyl transpeptidase; Hb: hemoglobin; HDL: high-density lipoprotein; Ig: immunoglobulin; INR: international normalized ratio; IQR: interquartile range; LDL: low-density lipoprotein; MCH: mean corpuscular hemoglobin; MCV: mean corpuscular volume; RDW: red cell distribution width; SD: standard deviation; TSH: thyroid-stimulating hormone; US: ultrasound; WBC: white blood cells

Supplementary Table S3: baseline (T0) CD immunological and histological features

	N (160)
AGA IgA (U/ml) (median; IQR)	36.0 (16.0-221.6)
Positive AGA IgA (n, %)	109 (68.1)
AGA IgG (U/ml) (median; IQR)	38.6 (19.5-106.4)
Positive AGA IgG (n, %)	120 (75.0)
tTg-IgA (U/ml) (median; IQR)	1163.0 (365.1-5146.6)
Positive tTg-IgA (n, %)	158 (98.8)
tTg-IgG (U/ml) (median; IQR)	62.5 (14.9-208.3)
Positive tTg-IgG (n, %)	110 (68.8)
Positive EMA (n, %)	159 (99.4)
HLA status:	
DQ2 positive (n, %)	146 (91.3)
DQ8 positive (n, %)	14 (8.8)
Subjects requiring EDS to confirm CD diagnosis (n, %)	26 (16.2*)
Marsh degree:	
3A (n, %)	8/26 (30.8)
3B (n, %)	8/26 (30.8)
3C (n, %)	10/26 (38.5)

* The remaining 134 subjects (83.8%) were diagnosed with the 'no biopsy-approach' according to the ESPGHAN criteria [11].

AGA: anti-gliadin antibody; CD: celiac disease; EDS: esophago-gastro-duodenoscopy; EMA: anti-endomysial antibody; ESPGHAN: European Society for Paediatric Gastroenterology Hepatology and Nutrition; Ig: immunoglobulin; IQR: interquartile range; SD: standard deviation; tTg: tissue transglutaminase

Supplementary Table S4: Clinical, laboratory and US features at follow-up (i.e. after 12 months of GFD) of CD population

	N (160)
Symptoms	
Asymptomatic at diagnosis (n, %)	27 (16.9)
Disappearance (n, %)	85 (53.1)
Reduction (n, %)	39 (24.4)
Unchanged (n, %)	8 (5.0)
Increase (n, %)	1 (0.6)
Biagi/Pavia GFD adherence score:	
Score 0 (n, %)	2 (1.3)
Score 1 (n, %)	1 (0.6)
Score 2 (n, %)	8 (5.0)
Score 3 (n, %)	23 (14.4)
Score 4 (n, %)	126 (78.8)
Hypertransaminasemia persistence (n, %)	7 (4.4)
US liver steatosis persistence (n, %)	3/99 (3.0)
AST (U/l) (mean ± SD)	24.1 (10.2)
ALT (U/l) (mean ± SD)	19.4 (8.3)

ALT: alanine aminotransferase; AST: aspartate aminotransferase; CD: celiac disease; GFD: gluten-free diet; SD: standard deviation; US: ultrasound

Supplementary Table S5: Comparison of CD immunological features at baseline (i.e. before start of GFD) and at follow-up (i.e. after 12 months of GFD)

	Before GFD (N=160)	After GFD (N=160)	P
AGA IgA (U/ml) (median; IQR)	36.0 (16.0-221.6)	9.5 (3.2-15.6)	<0.0001
Positive AGA IgA (n, %)	109 (68.1)	25 (15.6)	<0.0001
AGA IgG (U/ml) (median; IQR)	38.6 (19.5-106.4)	11.2 (4.2-15.6)	<0.0001
Positive AGA IgG (n, %)	120 (75.0)	27 (16.9)	<0.0001
tTg-IgA (U/ml) (median; IQR)	1163.0 (365.1-5146.6)	23.3 (12.1-113.8)	<0.0001
Positive tTg-IgA (n, %)	158 (98.8)	88 (55.0)	<0.0001
tTg-IgG (U/ml) (median; IQR)	62.5 (14.9-208.3)	5.0 (2.0-11.0)	<0.0001
Positive tTg-IgG (n, %)	110 (68.8)	34 (21.3)	<0.0001
Positive EMA (n,%)	159 (99.4)	9/21 (42.9)	<0.0001

AGA: anti-gliadin antibody; CD: celiac disease; EMA: anti-endomysial antibody; Ig: immunoglobulin; IQR: interquartile range; SD: standard deviation; tTg: tissue transglutaminase
